# Detection of Leptospiral DNA in the Urine of Donkeys on the Caribbean Island of Saint Kitts

**DOI:** 10.3390/vetsci4010002

**Published:** 2017-01-10

**Authors:** Bernard Grevemeyer, Michel Vandenplas, Brittney Beigel, Ellen Cho, Arve Lee Willingham, Ashutosh Verma

**Affiliations:** 1 Department of Clinical Sciences, Ross University School of Veterinary Medicine, P.O. Box 334, Basseterre 00265, St. Kitts; 2 Department of Biomedical Sciences, Ross University School of Veterinary Medicine, P.O. Box 334, Basseterre 00265, St. Kitts; mvandenplas@rossvet.edu.kn (M.V.); awillingham@rossvet.edu.kn (A.L.W.); 3 College of Veterinary Medicine, Lincoln Memorial University, 6965 Cumberland Gap Parkway, Harrogate, TN 37752, USA; brittney.beigel@lmunet.edu (B.B.); ellen.cho@lmunet.edu (E.C.)

**Keywords:** leptospirosis, donkey, zoonosis, waterborne disease

## Abstract

Leptospirosis is a global zoonosis caused by pathogenic spirochetes classified within the genus *Leptospira*. Leptospires live in the proximal renal tubules of reservoir or chronic carrier animals, and are shed in the urine. Naïve animals acquire infection either when they come in direct contact with a reservoir or infected animals or by exposure to environmental surface water or soil that is contaminated with their urine. In this study, urine samples from a herd of donkeys on the Caribbean island of St. Kitts were screened using a TaqMan-based real-time quantitative polymerase chain reaction (qPCR) targeting a pathogen-specific leptospiral gene, *lipl32*. Out of 124 clinically normal donkeys, 22 (18%) tested positive for leptospiral DNA in their urine. Water samples from two water troughs used by the donkeys were also tested, but were found to be free from leptospiral contamination. Detection of leptospiral DNA in the urine of clinically healthy donkeys may point to a role that these animals play in the maintenance of the bacteria on St. Kitts.

## 1. Introduction

Leptospirosis is an emerging disease in the United States, and a neglected disease in the rest of the world. Caused by pathogenic species of the genus *Leptospira*, leptospirosis is a public health threat that causes significant morbidity and mortality in animals and people. The spectrum of clinical presentations in human leptospirosis is very broad, ranging from a sub-clinical form to a potentially fatal syndrome involving multi-organ failure. Infection of domestic livestock results in significant losses due to spontaneous abortion, infertility, lowered milk production, and death [1,2]. Leptospirosis in dogs is frequently characterized by renal involvement, whereas equine leptospirosis is often associated with spontaneous abortion and recurrent uveitis [1,3].

Pathogenic leptospires live in the proximal renal tubules of reservoir animals and are shed in their urine, contaminating surface water. Environmental contamination by these reservoirs results in acquisition of infection by livestock and other domestic and wild animals. Maintenance of the disease in domestic animals is due to continued exposure to asymptomatic carriers or to transmission within herds. Humans are accidental hosts, and acquire the infection either when they come in direct contact with a reservoir or infected animals or by exposure to environmental surface water or soil that is contaminated with their urine. Leptospirosis is also an occupational threat to workers who are routinely exposed to open water sources or animals such as veterinarians, farmers, abattoir workers, meat inspectors, and rodent control workers. Natural disasters such as floods and hurricanes may be accompanied by leptospirosis outbreaks from contaminated water [4].

The Caribbean region is one of the major foci of leptospiral infection in the world [5]. Recently, we reported that around one-fifth of open-water sources on the island of St. Kitts have detectable leptospiral DNA [6]. In separate reports, we also described fatal cases of canine leptospirosis and renal carriage by a pig that was destined for human consumption [7]. Given the high incidence of leptospiral infection and involvement of multiple species, we tested urine from clinically normal donkeys at our teaching facility for the presence of leptospiral DNA using a highly sensitive TaqMan-based quantitative PCR that targets a pathogen-specific *lipl32* gene. To the best of our knowledge, this is the first report describing leptospiral urinary shedding in *Equidae* from any Caribbean country. 

## 2. Materials and Methods

### 2.1. Collection of Samples

**Figure 1:**
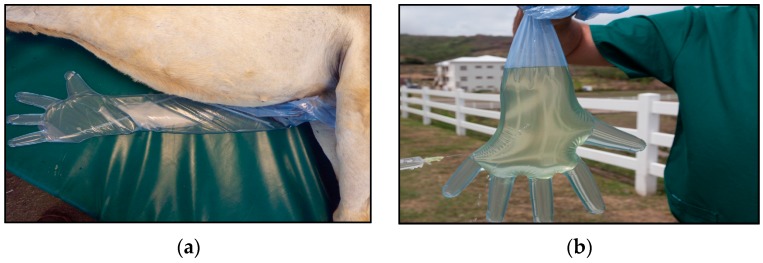
(**a**) Donkey under general anesthesia with palpation glove to collect urine; (**b**) Aspiration of urine sample into sterile syringe for collection.

Urine samples from 124 donkeys were collected during the seventh semester Large Animal Surgery labs. All animals received humane care in compliance with the “Principles of Laboratory Animal Care” formulated by the National Society for Medical Research and the “Guide for the Care and Use of Laboratory Animals” prepared by the National Institutes of Health. Clinical data were recorded from each donkey, which included age, gender, temperature, heart rate, respiratory rate, urine color, and examination of left and right eye. Donkeys were placed under general anesthesia, and urine was collected in palpation gloves (Figure 1). Ten milliliters of urine was collected from each donkey and centrifuged at 3000× *g* for 30 min at room temperature. The supernatant was aspirated and discarded. The remaining pellet was mixed by vortexing for 30 s and then frozen at −80 °C until DNA extractions were performed. Water from two troughs in the donkey pasture was collected and processed as described elsewhere [6].

### 2.2. DNA Extraction

DNA was extracted using DNeasy Blood and Tissue kit (Qiagen, Germantown, MD, USA). Samples were centrifuged as described above, and pellets were processed following the manufacturer’s instructions with some modifications [8].

*Leptospira interrogans* serovar Pomona was grown in Polysorbate-80 bovine serum albumin medium (NVSL) at 30 °C, and genomic DNA was isolated and quantified as previously described [9]. Based on the genome size of *L. interrogans* (4.659 Mb), genome equivalents were calculated as described in [10]. *L. interrogans* DNA concentration of 550 μg/mL was equivalent to ~1.13 × 10^11^ genome units/mL [10].

### 2.3. Quantitative Polymerase Chain Reaction (qPCR)

A TaqMan-based quantitative PCR targeting a 242 bp region of leptospiral *lipl32* gene was used to screen DNA extracted from the donkey urine, as described by Stoddard et al. [11]. The assay was performed in a MicroAmp Fast Optical 96-well reaction plate (Applied Biosystems) (Thermo Fisher Scientific corporation, Foster City, CA, USA). Each plate contained DNA equivalent to 10^7^, 10^6^, 10^5^, 10^4^, 10^3^, 10^2^, 10, and 1 leptospiral genome units. Each column (except positive control columns) had a no-template control. Each reaction was performed in a 25 μL final volume, using 5 μL of extracted DNA, 500 nM of LipL32-45F (forward primer; 5’-AAGCATTACCGCTTGTGGTG-3’), 500 nM of LipL32-286R (reverse primer; 5’-GAACTCCCATTTCAGCGATT-3’), and 100 nM of LipL32-189P (probe; FAM-5′-AAAGCCAGGACAAGCGCCG-3′-BHQ1) [11]. The assay was performed on a QuantStudio 3 Real-Time PCR system (Applied Biosystems) using Platinum Quantitative PCR SuperMix-UDG (Invitrogen) (Life Technologies Corporation, Carlsbad, CA, USA) and thermal conditions of a holding stage of 95 °C for 20 s, and 40 cycles of 95 °C for 3 s and 60 °C for 30 s.

## 3. Results and Discussion

The quantitative PCR used in this study targeted *lipl32*, which is a highly-conserved gene among pathogenic serovars encoding a 32 kDa major leptospiral membrane protein. This qPCR has previously been shown to be a robust assay for the detection of leptospiral DNA in clinical samples, and has high sensitivity and specificity [11].

**Figure 2:**
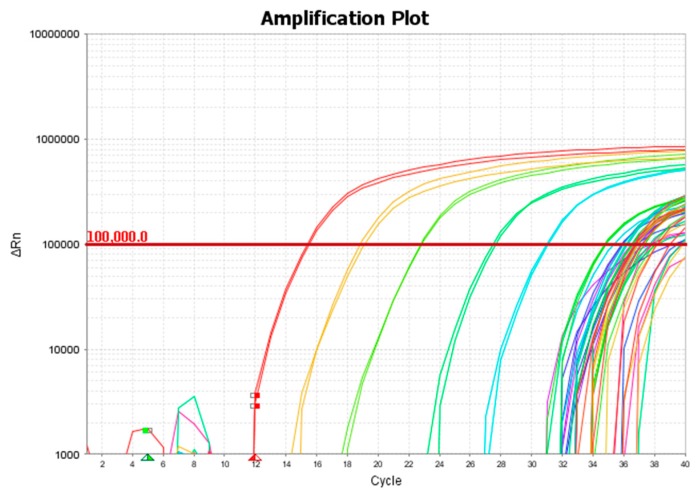
A representative amplification plot showing standards and positive samples.

All 124 donkeys were castrated males, and physical exams carried out on all donkeys were within normal limits on the day of collection. Temperatures were 99 °F ± 3 degrees, heart rates were between 35 and 60 bpm ± 2 beats, and respiratory rates were 8–30 bpm ± 2 breaths. Donkey ages ranged from 1 year old to mature adults. Urine color was clear/yellowish for all donkey samples, and both right and left eyes showed no obvious abnormalities. In addition, 300 mL water samples were collected from each of two water troughs located in the donkeys’ pasture. Samples were processed as described above, and extracted DNA was screened by qPCR. For analysis, a threshold of 100,000 and baseline between 5 and 12 cycles were used. Duplicate samples that had *Ct* < 40 were considered positive. Samples that were undetectable or had a *Ct* of > 40 were considered negative. In addition, a run was considered valid only if all ten no-template controls were negative. As described in our earlier work, a standard curve was obtained with 10^7^, 10^6^, 10^5^, 10^4^, 10^3^, 10^2^, 10 genome units of *L. interrogans* serovar Pomona (Rawlins). Out of 124 urine samples, 22 (18%) were positive for leptospiral DNA (Figure 2). Neither of the water samples were positive. 

The *Ct* values of positive samples ranged between 30 and 36, which is equivalent to approximately 8 × 10^4^ to 4 × 10^3^ genome units per 10 mL of urine. Although culturing of leptospires from urine was not attempted, our results indicate the shedding of leptospires in the urine of a significant number of tested animals. The infectious dose of leptospires is not known, but incidents of infection acquired through recreational water exposure suggest a low dose, so even low numbers of excreted organisms can be significant in the transmission and maintenance of the disease in an environment.

Clinical signs, duration of urinary shedding, and subsequent transmission is not well described in donkeys. Almost all of the information on leptospiral infection in donkeys comes from seroprevalence studies. In a recent leptospiral survey of domestic animals in Morocco, 20% of tested donkeys (3/15) were seropositive for *Leptospira* species [12]. Studies from Barbados and Trinidad—conducted more than three decades ago—showed a seroprevalence of 64% in horses and 76% in horses and donkeys, respectively [13,14]. Urinary shedding was not studied in any of these studies. Although studies from our group have described leptospirosis in other species on the island of St. Kitts, there is no information on leptospirosis in *Equidae*. Herein, we describe leptospiral shedding in the urine of donkeys. Detection of this zoonotic pathogen in the urine of clinically normal donkeys points to their role in maintenance of this disease on the island, and by extension, raises concerns about its public health implications.

A limitation of our current study is the use of only a single-target PCR, and that we did not test donkey sera for leptospiral exposure by MAT or attempted genotyping or culturing *Leptospira* spp. from the positive urine samples. In future studies, donkeys outside the institution should also be tested to determine infection status. Further testing with multiple time point sampling should be done to better understand duration of leptospiral shedding in this species.

## 4. Conclusions

In this study, we used a highly sensitive and specific quantitative PCR to detect leptospiral DNA in donkey urine. We found that 18% of the samples had detectable levels of leptospiral DNA. Evidence that clinically normal island donkeys shed leptospires in their urine should be used in disease awareness programs to minimize the risk of infection to humans and other animals.
